# Functional bony entrapment of the dorsalis pedis artery caused by cranial subluxation in the talonavicular joint

**DOI:** 10.1186/s42155-023-00410-w

**Published:** 2023-11-29

**Authors:** Karim Mostafa, Carmen Wolf, Matthias Bürger, Sebastian Kapahnke, Thorben Michaelis, Rouven Berndt, Rene Rusch, Julian Andersson, Philipp Jost Schäfer

**Affiliations:** 1 Department for Radiology and Neuroradiology, University Hospital Schleswig Holstein, Campus Kiel, Kiel, Germany; 2 Clinic of Cardiovascular Surgery, University Hospital Schleswig Holstein, Campus Kiel, Kiel, Germany

## Abstract

A 66-year-old female presented in the emergency department with Blue-Toe-Syndrome (BTS) and signs of osteitis of her left big toe. Imaging workup of the peripheral vasculature showed no findings. Upon invasive angiography, severe focal stenosis of the dorsalis pedis artery (DPA) could be seen at the talonavicular joint. Complete regression of the stenosis was inducible by dorsal extension in the ankle joint. Further imaging revealed an underlying subluxation of the talonavicular joint as cause of the arterial compression. Entrapment of the DPA is a rare condition and most often described in relation to connective tissue bands or variant muscular tendons (McCabe et al. 70:213–8, 2021; Weichman et al. 24:113, 2010; Smith et al.58:212–4, 2013; Griffin et al. 20:325–8; 2012). In the presented case, bony compression of the PDA due to cranial subluxation of the talus was seen as the cause of BTS and osteitis of the phalanx of the first toe.

## Introduction

Entrapment of the lower extremity vessels may lead to ischemia associated symptoms such as pain and intermittent foot claudication. In case of sole ischaemia of the digitus I, blue toe syndrome (BTS) may be present. BTS is defined as intermittent malperfusion of the big toe caused by vascular obstruction, resulting in pain and blue discoloration of the big toe. Studies report space-occupying lesions as underlying causes for this syndrome. One of the most commonly seen entrapment syndromes involves the popliteal artery, and here abnormally surrounding musculoskeletal structures such as the proximal part of the gastrocnemius muscle lead to functional arterial compression [1]. Cases of compression syndromes distal of the popliteal artery are rarely described, here Kaczynski et al. report a ganglion causing an entrapment of the posterior tibial artery [2]. Regarding entrapment of the DPA, anomalous fibrous tissue bands and an aberrant courses of the extensor hallucis brevis tendon are mentioned as possible causes [3–6]. To our knowledge, we describe the first case of intermittent osseous compression of the DPA by a subluxated talus during plantarflexion, causing BTS and osteitis.

## Case report

**Fig. 1:**
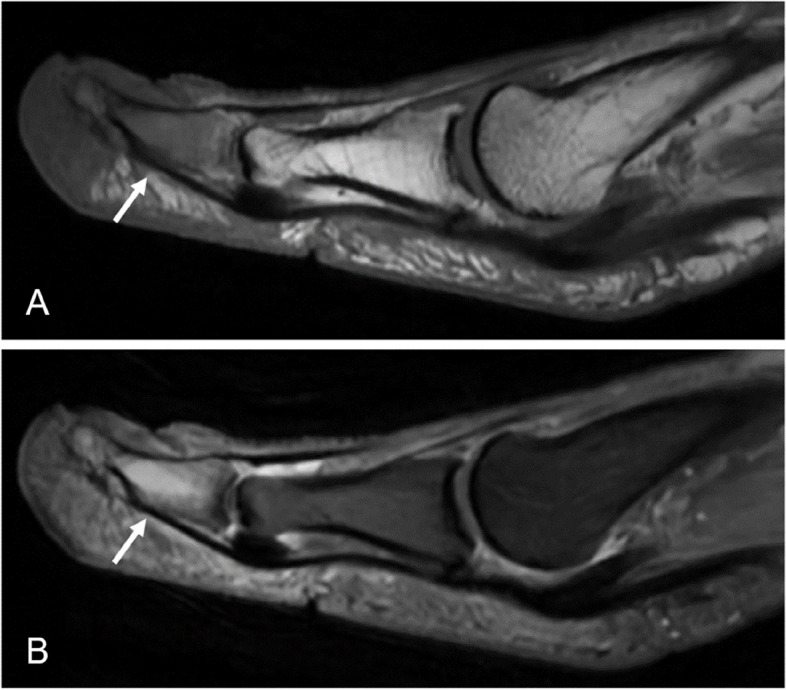
Initial magnetic resonance imaging of the phalanx of the first toe showing signs of osteomyelitis. In T1 weighted imaging loss of bone marrow signal in the distal phalanx of the digitus I can be seen (White arrow, Image **A**). In synopsis with hyperintense presentation of the affected segment in proton-density weighted imaging (White arrow, Image **B**) confirming bone edema, focal osteomyelitis was suspected

**Fig. 2:**
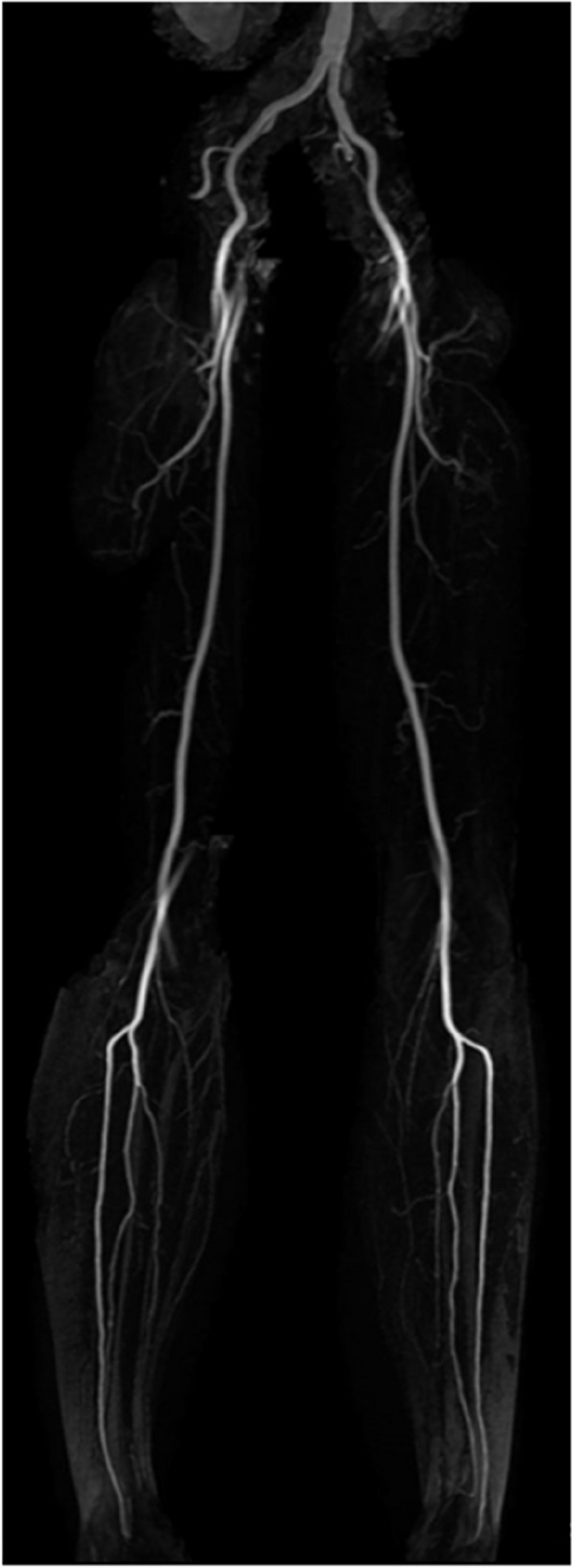
Magnetic resonance angiogram of the peripheral arteries in maximum - intensity - projection. Upon MR-angiography of the lower extremities, inconspicuous vascular anatomy was seen excluding vascular stenosis or atherosclerotic alterations down to the ankle level as cause of the pain in the left foot

A 66-year-old female presented with intermittent pain of the phalanx of the first toe for one year, especially while walking, increasingly limiting her mobility. Her past medical history included hypothyroidism adjusted by Levothyroxin, varices of the great saphenous vein treated by radiofrequency ablation 8 months priorly and an inguinal hernia which was minimally invasively repaired more than ten years ago. Upon clinical inspection, her big toe was seen to be swollen and of blue colour. No wounds or ulcerations were seen. Focussed imaging assessment of the left big toe was done with MRI, which showed intraosseous edema and loss of bone marrow signal suggesting osteitis (Fig. 1). Initially, an underlying ischemic cause was suspected, and the patient was referred to further imaging to rule out stenosis of the peripheral arterial system. Duplex sonography as well as contrast-enhanced MRI imaging was conducted, however, both imaging modalities showed no signs of reduced vascular perfusion. Upon MRI, there were clear signs of inflammation of the bone (Fig. 2).

**Fig. 3:**
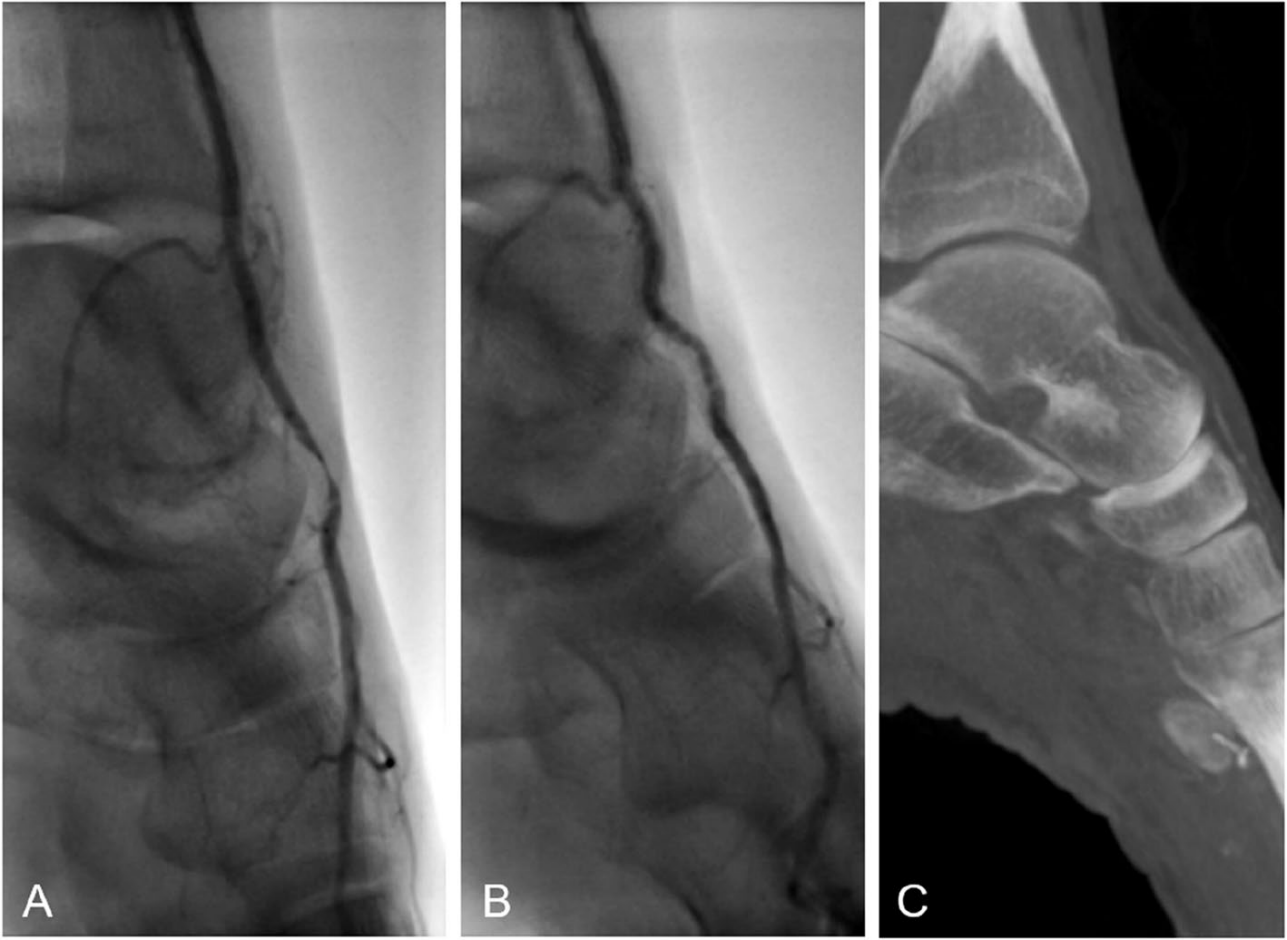
Dynamic fluoroscopic angiography imaging depicting DPA compression. In image **A**, an angiogram in spontaneous normal position of the left foot is seen with filiform stenosis of the DPA at the anterior cranial part of the talus. In image **B**, dynamic angiography in dorsal extension in the ankle joint shows complete disappearance of the stenosis, confirming functional vascular compression. Image **C** shows cone-beam CT imaging of the left foot depicting the talonavicular subluxation leading to functional intermittent bony compression of the DPA

Upon interdisciplinary review of the remaining symptoms and inconclusive diagnostic findings, indication for an invasive digital-subtraction-angiography (DSA) for imaging of the left-sided pedal arteries was set. The popliteal artery, the tibiofibular trunk as well as the fibular artery and the anterior and posterior tibial arteries showed normal contrast-media dynamics in DSA imaging. During the procedure, the patient developed vasospasms of the calf vasculature, which could be resolved with intra-arterial administration 0.2 mg nitroglycerin. Upon DSA imaging of the left ankle and foot, a relevant high-grade stenosis of the DPA could be clearly depicted with vessel diameter narrowing and contrast media stagnation. This finding was located precisely at the level of the talonavicular joint gap. Considering the unusual location for circumscribed atherosclerotic changes, dynamic DSA runs were performed with the patient’s foot firstly in neutral position and then in dorsal extension and plantar flexion of the ankle joint. In dorsal extension, the stenosis of the DPA was seen to resolve completely (Fig. 3). Subsequently, cone-beam computed tomography imaging was performed for anatomic visualization of the left foot. An abnormality was seen in the talonavicular articulation, whereby in its neutral position the distal part of the talus subluxated cranially creating a bony edge upon which subtotal compression of the DPA occurred. In fluoroscopy, this phenomenon could be aggravated by forced plantarflexion. In summary, these findings result in diagnosis of a functional bony compression of the DPA caused by the cranial edge of the talus due to a cranial subluxation in the talonavicular joint.

Interdisciplinary review of these findings was done by interventional radiologists, angiologists and vascular and orthopedic surgeons. After careful discussion with the patient on basis of shared decision making, no feasible interventional or operative treatment could be conceptualized. Conservative treatment with local vasodilatative ointment was initiated.

## Discussion

Among vascular compression syndromes, functional entrapment of the DPA is a rare condition. In the literature, only few cases of functional compression of the DPA are described, whereby compression was often present mainly in plantarflexion and located at different levels [3, 5]. One case of entrapment in dorsiflexion is also described [4]. In most cases, abnormal fibrous tissue bands or anatomic variants of the extensor hallucis brevis tendon were found as the cause of these compression syndromes [3–5]. In our patient, subluxation in the talonavicular articulation led to creation of an osseous ridge below the artery, causing it to be compressed in the neutral position of the ankle joint. In active plantarflexion, the subluxation increased, and the bony edge caused further compression of the DPA (Fig. 3). To our knowledge, this is the first ever description of osseous entrapment of the DPA.

Symptoms of BTS may be caused by many underlying conditions which are characterized by either decreased arterial inflow, impaired venous outflow or abnormal blood circulation [6, 7]. Mostly, BTS is traced back to atherosclerotic disease with pathomorphological alterations in the peripheral arteries, especially in elderly patients. This etiology was initially suspected in our case, however arterial disease was ruled out by MRI angiography down to the ankle level. Following DSA imaging allowed for functional assessment of the pedal arteries which ultimately pointed out the final diagnosis. If a vascular compression in the foot is suspected, it will remain crucial to perform diagnostic assessment with dynamic imaging, e.g. duplex ultrasound, MRI or DSA. In cases of DPA compression caused by fibrous tissue bands or muscle tendons, operative treatment may present a curative option. In the presented patient however, operative treatment was deemed unfeasible due to the likely needed larger-scale orthopaedic surgery. Surgically, incision of the tarsometatarsal joint and likely abrasion of the ventral aspect of the talus or osteosynthesis would have been necessary, and therefore it was agreed that the operative trauma and potential for complications were too big for the possible benefit. Further, there remained a risk of surgery causing forefoot instability.

Treatment of DPA entrapment is depending on its pathogenesis. In the cases presented by Weichmann et al. and Griffin et al., arterial compression was caused by the extensor hallucis brevis tendon, which could be resolved by transection and reinsertion of the tendon [4]. In the case presented by Smith et al., abnormal fibrous tissue bands causing intermittent compression were surgically dissected, leading to resolution of the patients’ symptoms [5].

## Conclusion

This report describes a functional osseous compression syndrome of the dorsalis pedis artery due to a subluxation in the talonavicular joint in a patient presenting with blue-toe-syndrome and forefoot pain. Functional arterial compression syndromes of the pedal arteries are a rare pathology, and this report is the first to describe an osseous pedal arterial compression as etiology.

## Abbreviations

BTS: Blue Toe Syndrome
DPA: Dorsalis Pedis Artery
